# Supplementary material for: Dpb11 facilitates the colocalization of Mec1-Ddc2 with its activators on gapped DNA
Source: Cell Rep. Author manuscript; Available in PMC 2026 Sep 3. (PMC13539869; doi:10.1016/j.celrep.2026.117660)
Supplement: Supplementary file 1 [file NIHMS2199962-supplement-1.pdf]

Cell Reports, Volume *45*

Supplemental information

Dpb11 facilitates the colocalization of Mec1-Ddc2 with its activators on gapped DNA

Emily C. Beckwitt, Gabriella N.L. Chua, Shixin Liu, and Michael E. O'Donnell

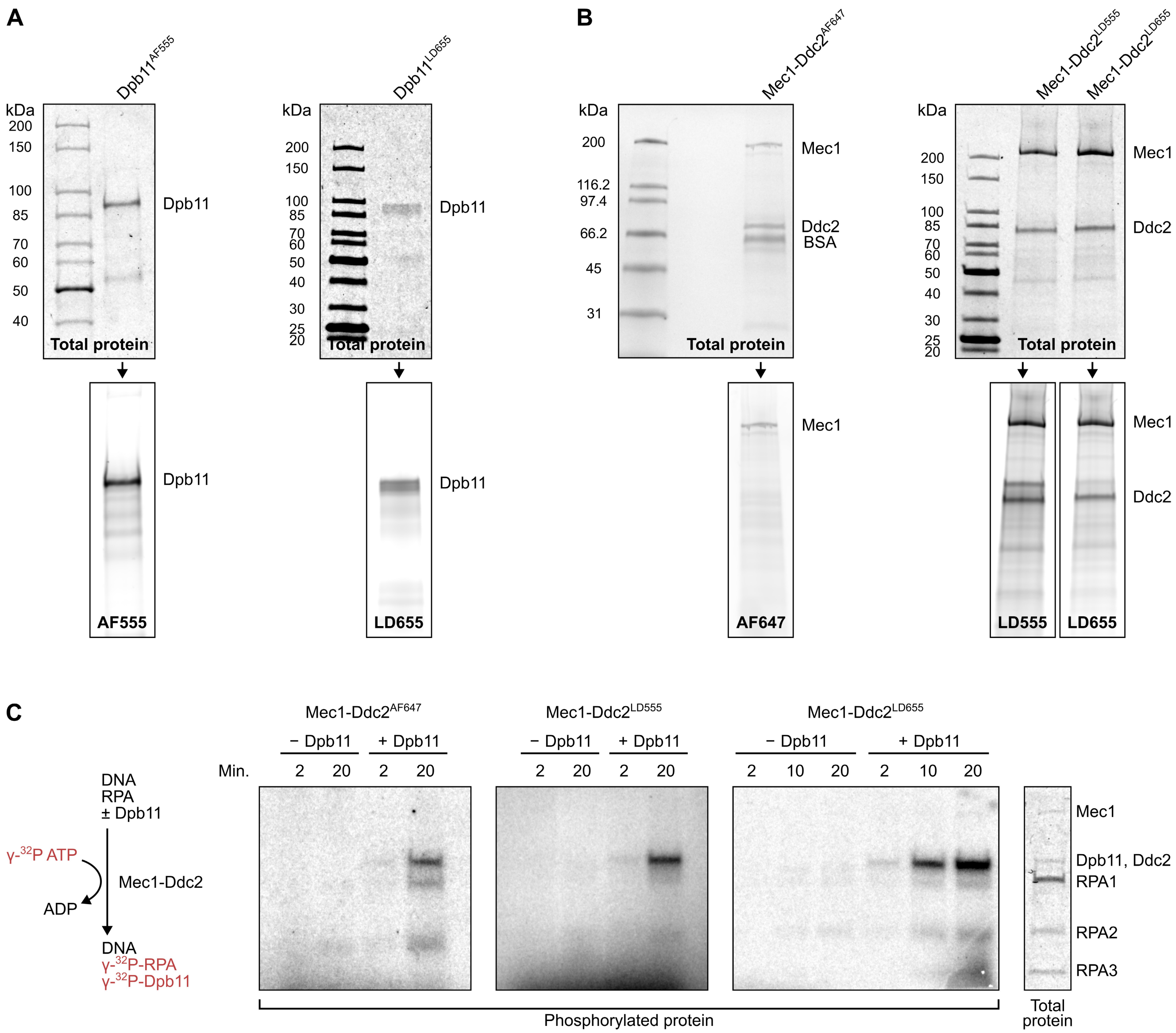

Figure S1. Proteins used in this study. (A) SDS-PAGE of purified Dpb11^{AF555} and Dpb11^{LD655}, stained for total protein (top) and scanned for fluorescence (bottom). (B) SDS-PAGE of purified Mec1-Ddc2^{AF647}, Mec1-Ddc2^{LD555}, and Mec1-Ddc2^{LD655}, stained for total protein (top) and scanned for fluorescence (bottom). (C) Phosphorylation assay to test Dpb11 activation of Mec1 kinase. Purified and fluorescently labeled Mec1-Ddc2 was incubated with ATP, 18-primed ΦX174 ssDNA, and RPA, with or without Dpb11 for the indicated times. Phosphorylation reactions were separated by SDS-PAGE, stained for total protein (right), and phosphorimaged to detect incorporation of γ-^{32}P ATP.

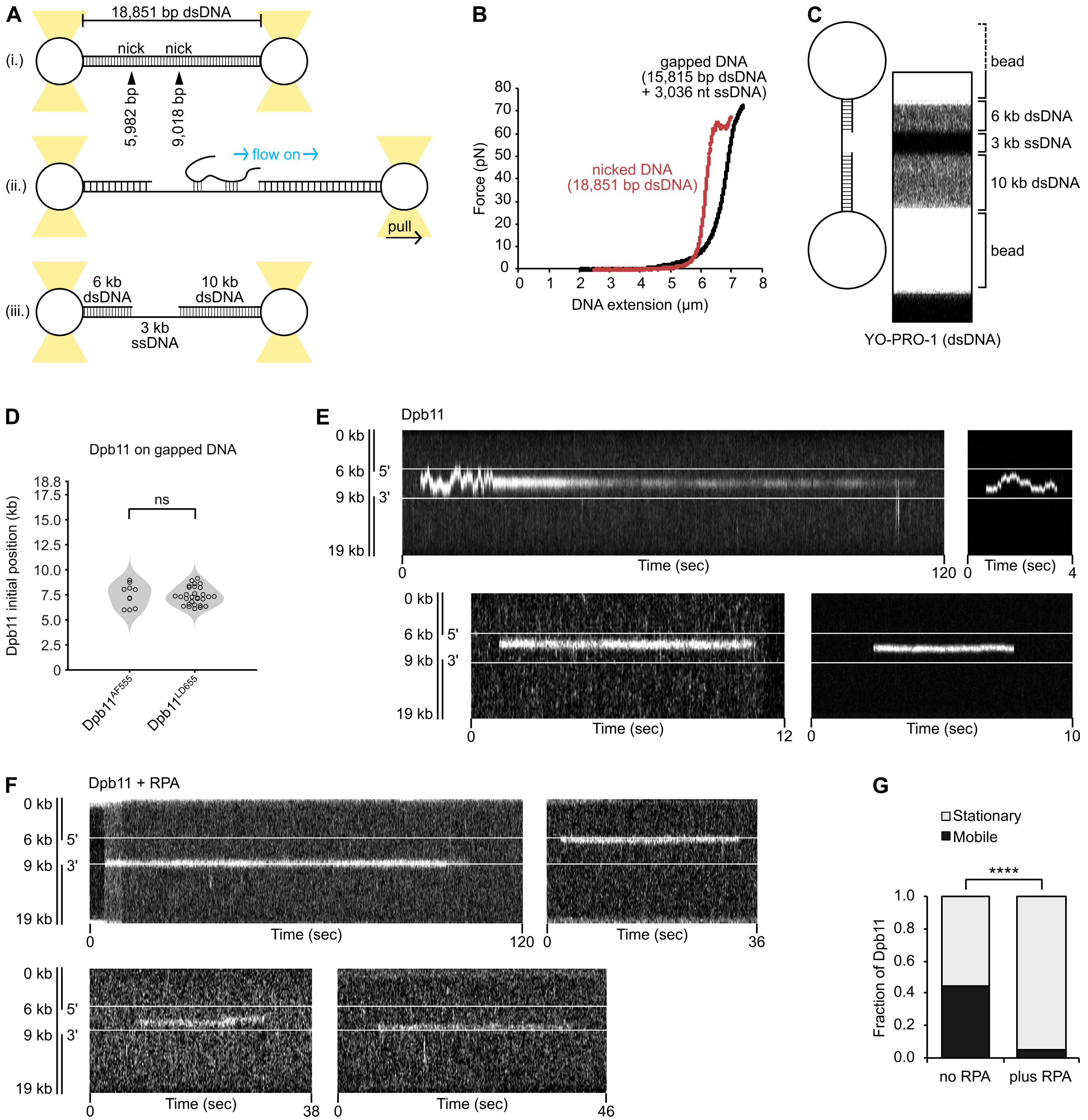

Figure S2. Single-molecule imaging of Dpb11 on gapped DNA, related to Figure 1. (A) Generation of 3 kb-gapped DNA for single-molecule experiments. (B) Sample experimental data confirming the change in force-extension curves before (red) and after (black) generation of the ssDNA gap. (C) Kymograph (15 sec) showing YO-PRO-1 staining of dsDNA on 3 kb-gapped DNA substrate. (D) Comparison of DNA-binding behavior of Dpb11 labeled with AF555 and LD655. Samples were compared by Mann Whitney U test. ns, not significant. (E and F) Additional kymographs of $Dpb11^{AF555}$ and $Dpb11^{LD655}$ on 19 kb DNA with 3 kb single-stranded gap (6–9 kb) in the absence (E) or presence (F) of RPA. See Figures 1B and 1D, respectively. (G) Fraction of stationary and mobile Dpb11 particles on 3 kb-gapped DNA in the absence or presence of RPA. **** $p = 6.8\times10^{-5}$ by χ^2 test for independence.

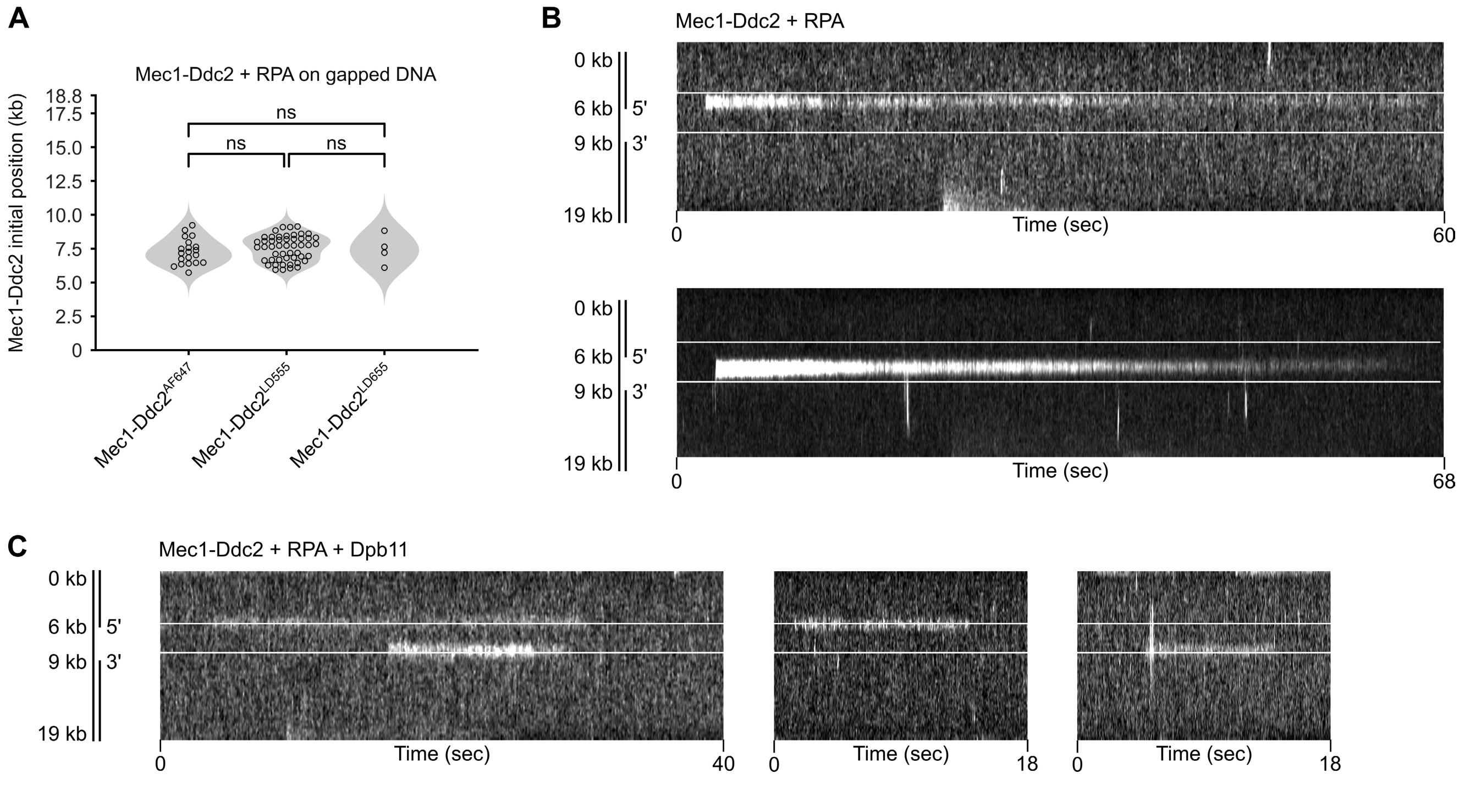

Figure S3. Single-molecule imaging of Mec1-Ddc2 on gapped DNA, related to Figure 2. (A) Comparison of DNA-binding behavior of Mec1-Ddc2 labeled by AF647, LD555, and LD655. Samples were compared by Mann Whitney U test. ns, not significant. (B and C) Additional kymographs of Mec1-Ddc2^{AF647}, Mec1-Ddc2^{LD655}, and Mec1-Ddc2^{LD555} with unlabeled RPA on 19 kb DNA with 3 kb single-stranded gap (6–9 kb) in the absence (B) or presence (C) of Dpb11. See Figures 2A and 2C, respectively.

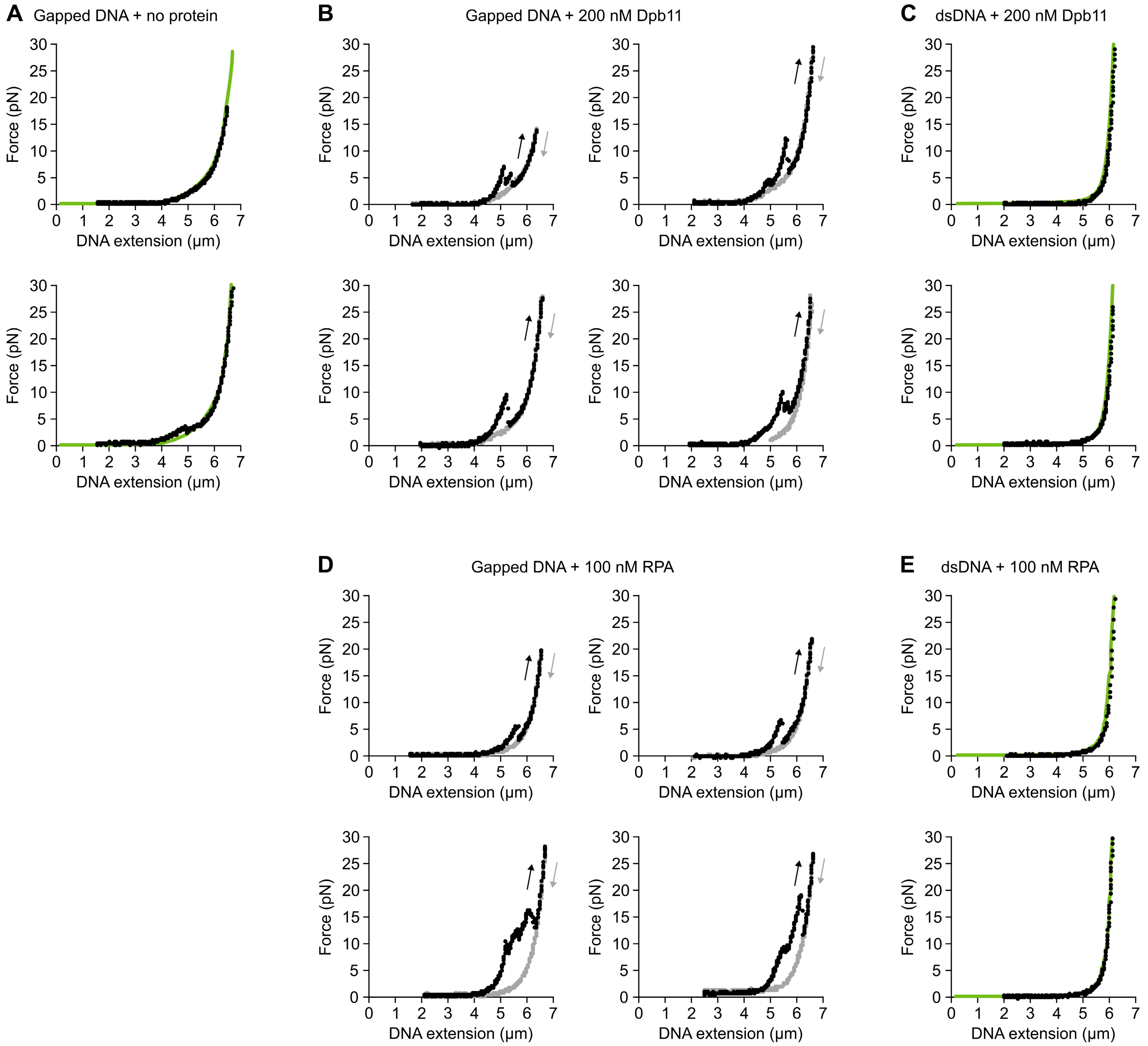

Figure S4. DNA pulling tests, related to Figure 3. (A) Example DNA pulling tests of gapped DNA (15.815 kb dsDNA + 3.036 kb ssDNA) in the absence of proteins. Black circles are experimental data. Green line is the predicted hybrid worm-like chain (WLC) + freely-jointed chain (FJC) model (Figure S7B). (B) Additional example DNA pulling tests of gapped DNA in the presence of Dpb11. Black circles are stretching data and gray circles are relaxation data. See Figure 3B. (C) Example DNA pull tests of nicked DNA (18.851 kb dsDNA) in the presence of Dpb11. Black circles are experimental data. Green line is the predicted WLC model (Figure S7A). (D) Example DNA pull tests of gapped DNA in the presence of RPA. See Figure 3E. (E) Example DNA pull tests of nicked DNA in the presence of RPA. Black circles are experimental data. Green line is the predicted WLC model (Figure S7A).

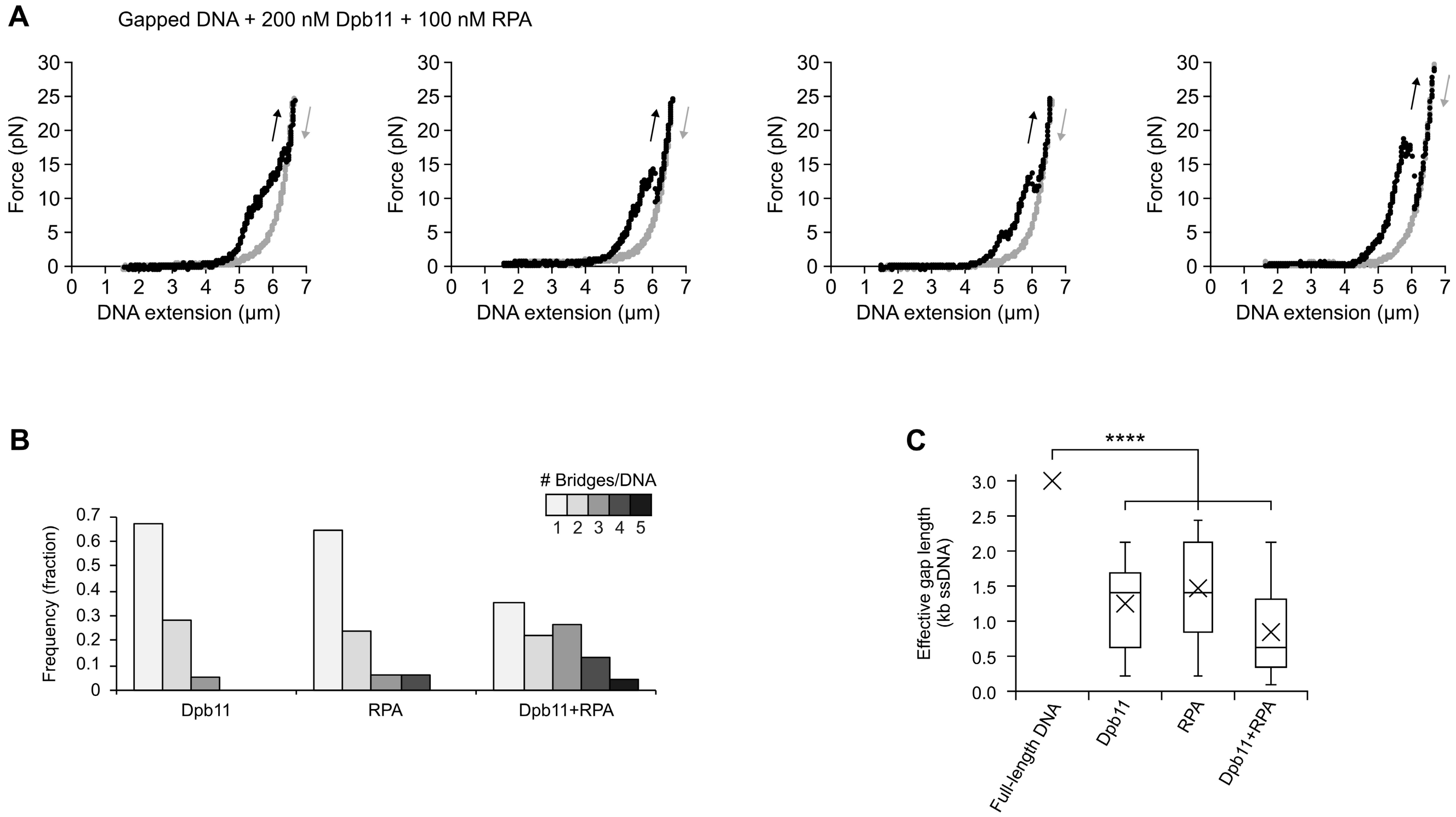

Figure S5. Dpb11 bridges gapped DNA in the presence of RPA, related to Figure 3. (A) Additional example DNA pulling tests of 3 kb-gapped DNA in the presence of Dpb11 + RPA. Black circles are stretching data and gray circles are relaxation data. See Figure 3H. (B) Histograms showing the number of bridges per DNA molecule in the presence of Dpb11 only, RPA only, or both Dpb11+RPA. (C) The effective gap length (3.036 kb – sum of all loop lengths) in the presence of Dpb11 only, RPA only, or both Dpb11+RPA. **** $p < 1.0 \times 10^{-7}$ by one sample t-tests for all three conditions compared to full-length gapped DNA (3.036 kb ssDNA).

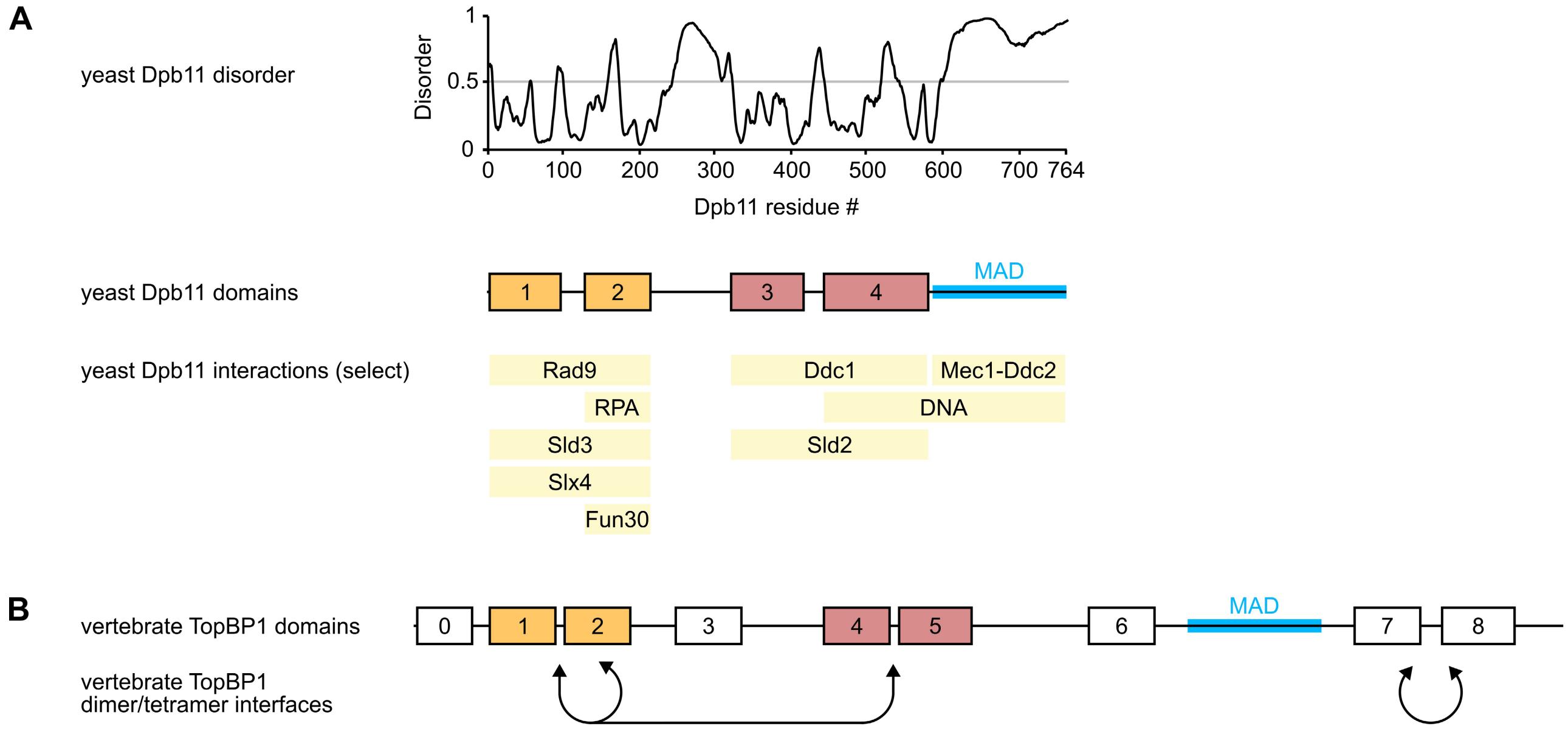

Figure S6. Dpb11 domains, interaction partners, and homology, related to Figure 4. (A) Disorder predictions for yeast Dpb11 were obtained via the PONDR VSL2 algorithm[1]. BRCA1 C-terminal (BRCT) domains (numbered rectangles) and the Mec1-activating domain (MAD, blue) are shown below the disorder predictions. Select Dpb11 binding partners are displayed according to their respective interaction sites on Dpb11. Interactions involving DNA damage checkpoint activation: Rad9[2], Ddc1[3,4], Mec1-Ddc2[2,5,6]. Interactions with DNA and RPA, reported previously[7,8] and further investigated in this study. Interactions involved in other pathways: Sld2 and Sld3[9-12], Slx4[13,14], Fun30[15]. (B) Vertebrate TopBP1 BRCT domains (numbered rectangles) and Mec1/ATR activating domain (MAD, blue) are shown. Domains are colored according to their homologous counterparts in yeast. Self-oligomerization interfaces are indicated below domains[16].

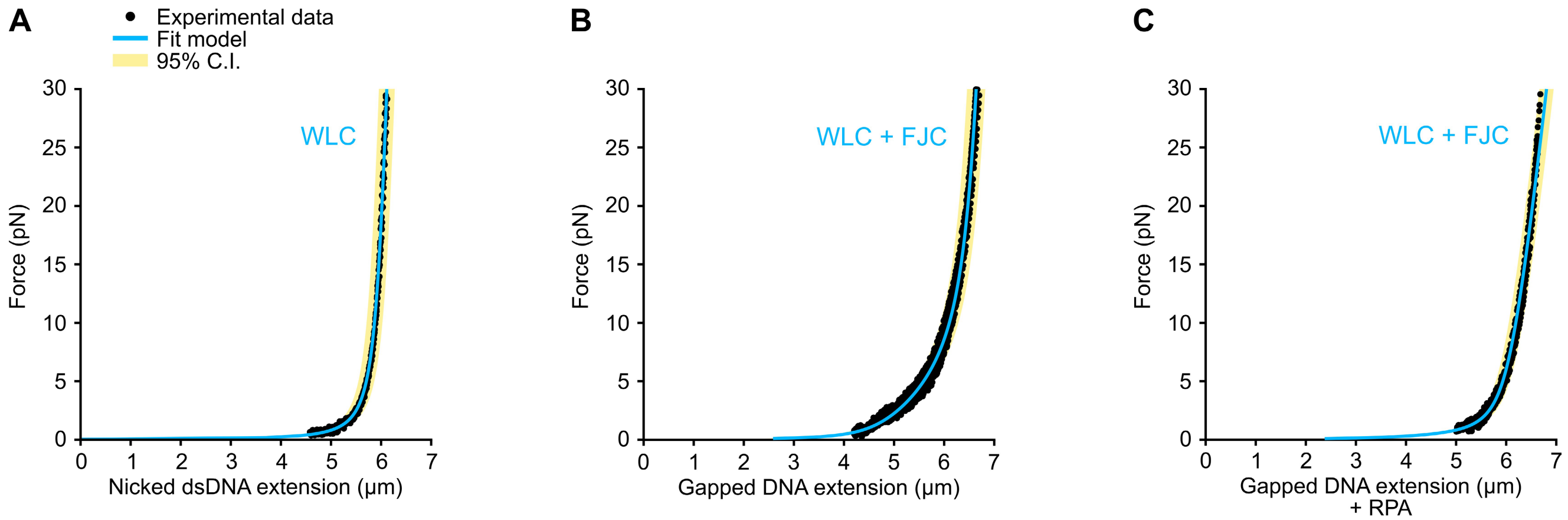

Figure S7. DNA polymer modeling. Force-extension data for 18.851 kb DNA substrates in the absence of proteins were globally fit by least squares nonlinear regression to the worm-like chain model (WLC) or hybrid worm-like chain and freely-jointed chain model (WLC + FJC). Black circles: experimental data. Blue line: fit model. Yellow shading: 95% confidence interval of fit. (A) Nicked dsDNA (18,851 bp dsDNA) fit to WLC model (n = 4 DNA molecules). (B) Gapped DNA (15,815 bp dsDNA + 3,036 nt ssDNA) fit to WLC+FJC model (n = 6 DNA molecules). (C) Gapped DNA (15,815 bp dsDNA + 3,036 nt ssDNA) in the presence of RPA fit to WLC+FJC model (n = 6 DNA molecules).

SUPPLEMENTAL REFERENCES

1. Peng, K., Radivojac, P., Vucetic, S., Dunker, A.K., and Obradovic, Z. (2006). Length-dependent prediction of protein intrinsic disorder. BMC Bioinformatics *7*, 208. 10.1186/1471-2105-7-208.
2. Pfander, B., and Diffley, J.F. (2011). Dpb11 coordinates Mec1 kinase activation with cell cycle-regulated Rad9 recruitment. EMBO J *30*, 4897-4907. 10.1038/emboj.2011.345.
3. Wang, H., and Elledge, S.J. (2002). Genetic and physical interactions between DPB11 and DDC1 in the yeast DNA damage response pathway. Genetics *160*, 1295-1304. 10.1093/genetics/160.4.1295.
4. Puddu, F., Granata, M., Di Nola, L., Balestrini, A., Piergiovanni, G., Lazzaro, F., Giannattasio, M., Plevani, P., and Muzi-Falconi, M. (2008). Phosphorylation of the budding yeast 9-1-1 complex is required for Dpb11 function in the full activation of the UV-induced DNA damage checkpoint. Mol Cell Biol *28*, 4782-4793. 10.1128/MCB.00330-08.
5. Mordes, D.A., Nam, E.A., and Cortez, D. (2008). Dpb11 activates the Mec1-Ddc2 complex. Proc Natl Acad Sci U S A *105*, 18730-18734. 10.1073/pnas.0806621105.
6. Navadgi-Patil, V.M., and Burgers, P.M. (2008). Yeast DNA replication protein Dpb11 activates the Mec1/ATR checkpoint kinase. J Biol Chem *283*, 35853-35859. 10.1074/jbc.M807435200.
7. Dhingra, N., Bruck, I., Smith, S., Ning, B., and Kaplan, D.L. (2015). Dpb11 protein helps control assembly of the Cdc45.Mcm2-7.GINS replication fork helicase. J Biol Chem *290*, 7586-7601. 10.1074/jbc.M115.640383.
8. Bruck, I., Dhingra, N., Martinez, M.P., and Kaplan, D.L. (2017). Dpb11 may function with RPA and DNA to initiate DNA replication. PLoS One *12*, e0177147. 10.1371/journal.pone.0177147.
9. Zegerman, P., and Diffley, J.F. (2007). Phosphorylation of Sld2 and Sld3 by cyclin-dependent kinases promotes DNA replication in budding yeast. Nature *445*, 281-285. 10.1038/nature05432.
10. Tanaka, S., Umemori, T., Hirai, K., Muramatsu, S., Kamimura, Y., and Araki, H. (2007). CDK-dependent phosphorylation of Sld2 and Sld3 initiates DNA replication in budding yeast. Nature *445*, 328-332. 10.1038/nature05465.
11. Kamimura, Y., Masumoto, H., Sugino, A., and Araki, H. (1998). Sld2, which interacts with Dpb11 in Saccharomyces cerevisiae, is required for chromosomal DNA replication. Mol Cell Biol *18*, 6102-6109. 10.1128/MCB.18.10.6102.
12. Tak, Y.S., Tanaka, Y., Endo, S., Kamimura, Y., and Araki, H. (2006). A CDK-catalysed regulatory phosphorylation for formation of the DNA replication complex Sld2-Dpb11. EMBO J *25*, 1987-1996. 10.1038/sj.emboj.7601075.
13. Ohouo, P.Y., Bastos de Oliveira, F.M., Almeida, B.S., and Smolka, M.B. (2010). DNA damage signaling recruits the Rtt107-Slx4 scaffolds via Dpb11 to mediate replication stress response. Mol Cell *39*, 300-306. 10.1016/j.molcel.2010.06.019.
14. Gritenaite, D., Princz, L.N., Szakal, B., Bantele, S.C., Wendeler, L., Schilbach, S., Habermann, B.H., Matos, J., Lisby, M., Branzei, D., and Pfander, B. (2014). A cell cycle-regulated Slx4-Dpb11 complex promotes the resolution of DNA repair intermediates linked to stalled replication. Genes Dev *28*, 1604-1619. 10.1101/gad.240515.114.
15. Bantele, S.C., Ferreira, P., Gritenaite, D., Boos, D., and Pfander, B. (2017). Targeting of the Fun30 nucleosome remodeller by the Dpb11 scaffold facilitates cell cycle-regulated DNA end resection. Elife *6*. 10.7554/eLife.21687.
16. Kim, A., Montales, K., Ruis, K., Senebandith, H., Gasparyan, H., Cowan, Q., and Michael, W.M. (2020). Biochemical analysis of TOPBP1 oligomerization. DNA Repair (Amst) *96*, 102973. 10.1016/j.dnarep.2020.102973.
